# Transcriptional regulation of dendritic cell development and function

**DOI:** 10.3389/fimmu.2023.1182553

**Published:** 2023-07-14

**Authors:** Shengbo Zhang, Cindy Audiger, Michaël Chopin, Stephen L. Nutt

**Affiliations:** ^1^Immunology Division, Walter and Eliza Hall Institute of Medical Research, Parkville, VIC, Australia; ^2^Department of Medical Biology, University of Melbourne, Parkville, VIC, Australia; ^3^Department of Biochemistry and Molecular Biology, Biomedicine Discovery Institute, Monash University, Clayton, VIC, Australia

**Keywords:** dendritic cells, transcription factor, IRF8, cDCs, pDCs

## Abstract

Dendritic cells (DCs) are sentinel immune cells that form a critical bridge linking the innate and adaptive immune systems. Extensive research addressing the cellular origin and heterogeneity of the DC network has revealed the essential role played by the spatiotemporal activity of key transcription factors. In response to environmental signals DC mature but it is only following the sensing of environmental signals that DC can induce an antigen specific T cell response. Thus, whilst the coordinate action of transcription factors governs DC differentiation, sensing of environmental signals by DC is instrumental in shaping their functional properties. In this review, we provide an overview that focuses on recent advances in understanding the transcriptional networks that regulate the development of the reported DC subsets, shedding light on the function of different DC subsets. Specifically, we discuss the emerging knowledge on the heterogeneity of cDC2s, the ontogeny of pDCs, and the newly described DC subset, DC3. Additionally, we examine critical transcription factors such as IRF8, PU.1, and E2-2 and their regulatory mechanisms and downstream targets. We highlight the complex interplay between these transcription factors, which shape the DC transcriptome and influence their function in response to environmental stimuli. The information presented in this review provides essential insights into the regulation of DC development and function, which might have implications for developing novel therapeutic strategies for immune-related diseases.

## 1 Introduction

Our body is constantly exposed to danger in the form of pathogenic micro-organisms that seek to break through the skin and the mucous membranes that provide the first barrier of defense. The acquisition of mutations in our own cells resulting in their transformation into malignant clones represents another form of danger to which the body must respond in order to avoid the development of cancer. A rare group of heterogeneous immune cells known collectively as dendritic cells (DCs) are central to sensing these dangers and orchestrating the appropriate response, while at the same time ignoring normal healthy cells and commensal micro-organisms.

DCs are a diverse group of cell types that are widely dispersed throughout the body. They act as sentinels to capture exogenous antigens that are processed and presented via either major histocompatibility complex class II (MHC-II) to CD4^+^ T cells (direct presentation) or shuttled through a specialized pathway to MHC-I to engage CD8^+^ T cells (cross-presentation) (1–5). Antigen uptake alone is insufficient to fully activate DCs, thus allowing DCs to remain tolerant to harmless antigens derived from healthy tissue or commensal microbes (6–12). However, DCs express an array of pattern-recognition receptor (PRRs) and C-type lectin receptors (CLRs) whose engagement induces maturation and migration, key steps in promoting their interaction with antigen specific T cells and thereby initiating adaptive immunity (13–15).

To face this variety of immune challenges, DCs have evolved into a variety of phenotypically and functionally distinct cellular subsets in both mouse and human (5, 16–19). DCs can be broadly separated into conventional dendritic cells (cDCs), plasmacytoid DCs (pDCs), and monocyte-derived DCs (moDCs), the latter becoming prevalent during inflammation. Conventional DCs can be further divided into type 1 cDC (cDC1s) and type 2 cDCs (cDC2s). Of note Langerhans Cells that were traditionally classified as DCs due to their morphological and phenotypic similarities with DCs and their ability to prime T cell response, are now recognized to be a specialized population of tissue macrophages (20, 21), and therefore their ontogenetic and homeostatic properties differ greatly from DC (22, 23).

**Figure 1:**
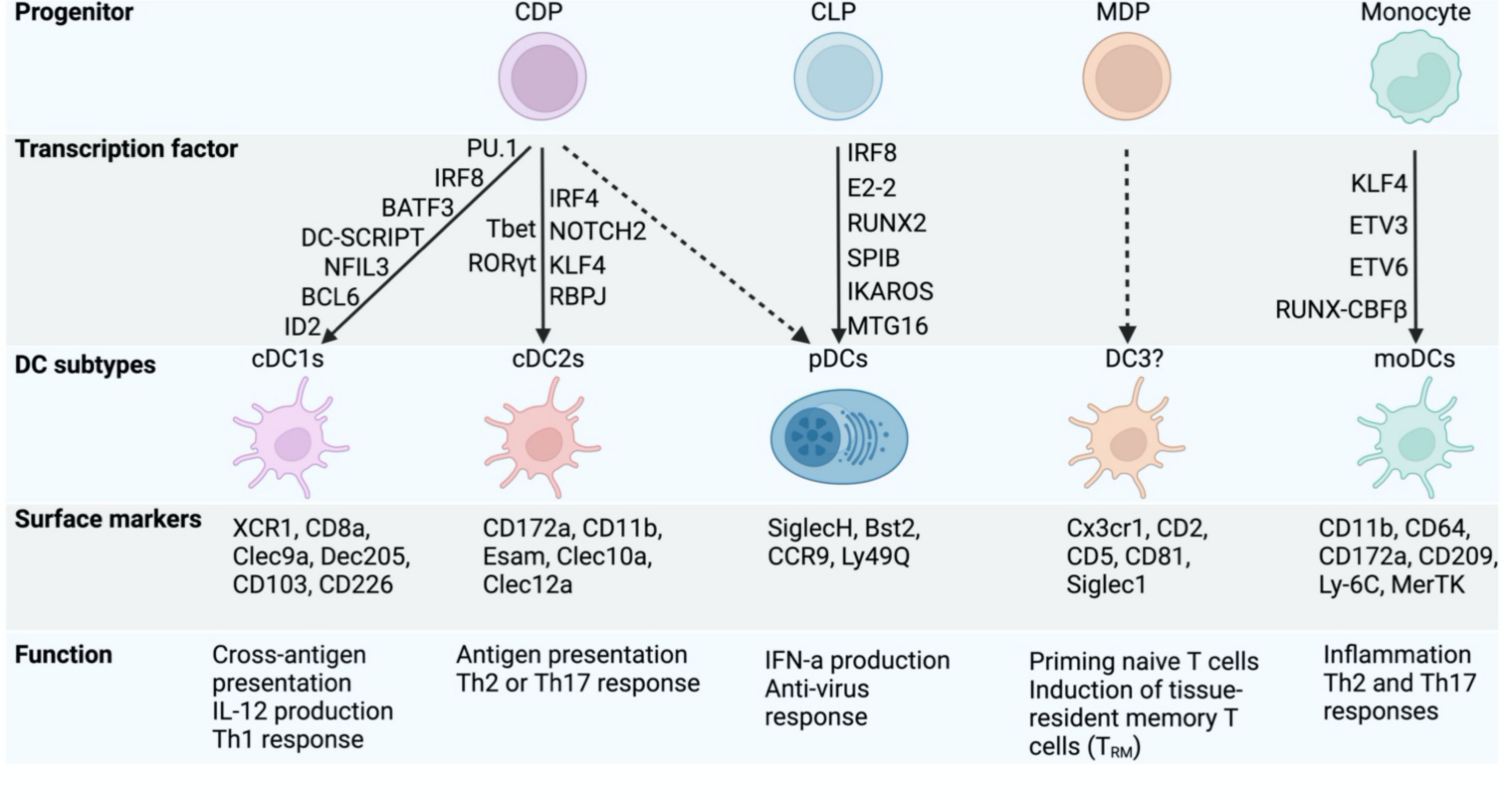
Transcription factors controlling DC specification and function. The figure highlights the development of cDCs subtypes and pDCs from the shared common dendritic cell progenitors (CDP). Some other DCs subtypes (DC3) have also been reported recently in mouse and human and derive from monocyte-dendritic cell progenitors (MDP). The common lymphoid progenitor (CLP) generates pDCs but lack cDC potential. Under inflammation, monocytes can differentiate into monocyte-derived (mono)DCs. Each DC subtype has unique surface markers and attributes in regulating immune response. The transcription factors governing DC lineage specification and function are shown.

Generally, mouse cDCs and moDCs are defined by high cell surface expression of the integrin CD11c (encoded by *Itgax*) and MHC-II. Beyond the expression of CD11c and MHC-II, additional cell surface markers can be used to distinguish mouse DC subsets. cDC1s co-express the cell surface molecules XCR1, CD24, DEC205, CD8a and CLEC9A (24, 25) (**Figure 1**). In the peripheral lymphoid and non-lymphoid organs such as the lung, gut and LN, cDC1s also can also be identified as CD103^+^CD11b^-^ cDCs (26, 27). The splenic cDC2 subset is defined by the presence of CD11b, Sirpα (CD172a) and CD4 on the cell surface (28, 29). Adding to that cDC2s can co-express CD103^+^CD11b^+^ in non-lymphoid organs (27, 30). Although the cDC2 compartment has been described as a discrete subset, the advent of single cell technology has revealed a high degree of diversity within this population and some additional markers have been proposed to define the basis of this heterogeneity (discussed later). Under inflammatory conditions, moDCs can respond to the chemokines such as CCL2 and CCL7 and upregulate cell surface expression of MHC-II, CD11c and CD11b, and thus can be easily mistaken as cDC2s (31). Additional markers such as CD64 and MAR-1 can be used to discriminate moDCs from cDC2s (32). pDCs are distinct from the other DC subsets in that they exhibit a lower level of expression of CD11c and MHC-II. pDCs also express a variety of unique markers (compared to cDCs and moDCs), including BST2, B220, and SiglecH (33). Whether pDC belongs to the DC lineage remains at present a matter of debate given that pDCs express some lymphoid markers and overall have a limited capacity to present antigens to T cells compared to the cDC or moDC compartments (34–36).

Given their critical role in orchestrating adaptive immune responses, high dimensional and throughput techniques such as single cell RNAseq and Cytometry by time of flight (CyTOF), have been applied to the DC lineages. These approaches have revealed unexpected heterogeneity within the DC subsets in both mouse and human, especially the cDC2s (37–39). Single-cell analysis of human mononuclear phagocytes also identified an inflammatory subset of CD5^-^CD163^+^CD14^+^ inflammatory DC3s that were distinct from cDC2s and able to prime Th2 responses (40). The integration of these newly identified subsets into the overall picture of DC development is a very active area of current research (41–43). In this review we will focus on the recent insights on both the transcriptional programming and the ontogeny of the DC lineages and discuss how these findings inform our understanding of the functional specialization of the DC subsets.

## 2 cDC1 development and function

### 2.1 Transcriptional regulation of cDC1 development

cDC1s differentiate principally from the common dendritic cell progenitor (CDP), a population that also gives rise to cDC2s (44, 45). A CDP subset committed to cDC1 fate has been characterized through the expression of CD11c^–^MHC-II^-/int^CD117^int^Zbtb46-GFP^+^ in the bone marrow (46) and pre-cDC1s (CD11c^+^MHC-II^-/int^CD135^+^CD172^-^Siglec-H^-^Ly6C^-^) (47) in the bone marrow and spleen (44, 48, 49). However, cellular barcoding and fate mapping studies have challenged this linear model of differentiation, given that cDC1 imprinting could be detected as early as the hematopoietic stem cell (HSC) (50–52).

Despite the challenges surrounding their origin, there is a very good understanding of the transcriptional mechanisms controlling cDC1 differentiation. cDC1 commitment is dependent on the expression of specific transcription factors (TFs), including BATF3 (Basic Leucine Zipper ATF-Like Transcription Factor 3) (53), IRF8 (Interferon Regulatory Factor 8) (54), PU.1 (55), NFIL3 (Nuclear Factor, Interleukin 3 Regulated) (56, 57), and ID2 (Inhibitor of DNA Binding 2) (58), where the specific inactivation of any of these TFs is associated with a strong defect in cDC1 development (**Figure 1**). However, this cDC1 deficiency can be rescued by short-term bone marrow reconstitution (59) or over-expressing IRF8 in absence of BATF3 (60), highlighting the significant role of IRF8 and the fine network of TFs allowing cDC1 differentiation.

**Figure 2:**
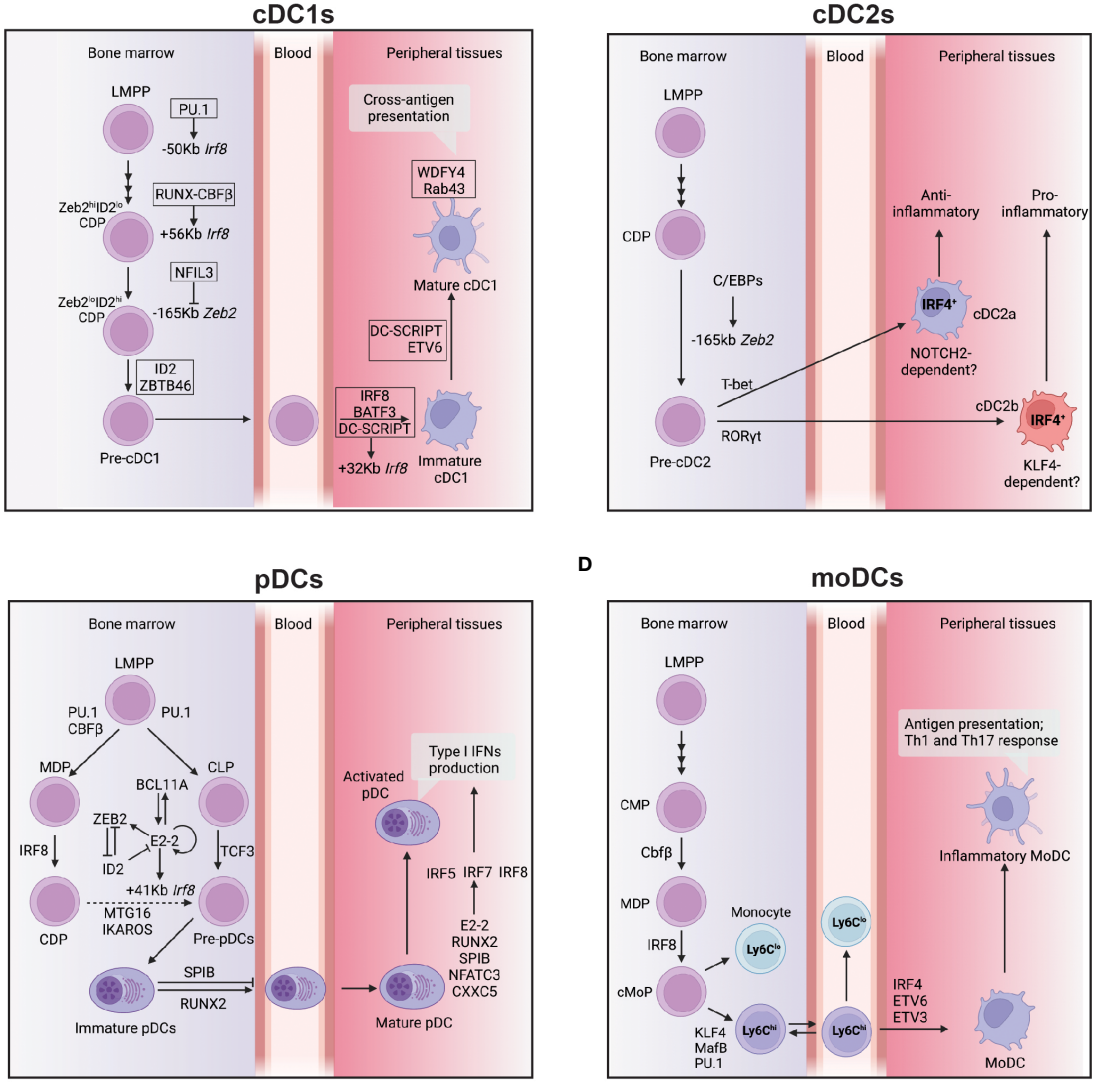
Transcriptional network controlling the development and function of DCs subsets. **(A)** This figure depicts the transcriptional network that regulates the development and function of cDC1s from bone marrow progenitors to peripheral tissues. The transcription factor IRF8 plays a crucial role in cDC1 development, and its expression is regulated by several enhancers located at -50 kb, +56 kb, and +32 kb relative to the *Irf8* gene locus. The transcription factors PU.1, RUNX-CBFβ, BATF3, and DC-SCRIPT activate these enhancers at different stages of cDC1 development. In addition, NFIL3 is required for cDC1 development, and it suppresses ZEB2 expression via binding at-165kb *Zeb2* enhancer during the CDP stage. ZBTB46 expression marks the commitment to the cDC1 lineage, while DC-SCRIPT and ETV6 promote the maturation process of cDC1s. WDFY4 is a co-activator that primarily controls the cross-antigen presentation ability of mature cDC1s. **(B)** cDC2s express IRF4 and can be further divided into two subtypes: cDC2a, which have an anti-inflammatory function, and cDC2b, which have a pro-inflammatory function. The development of cDC2a requires T-bet, while the development of cDC2b requires RoRγt. Both cDC2a and cDC2b develop from a CDP and this process is controlled by C/EBP binding at the -165kb zeb2 enhancer. **(C)** This figure illustrates the transcriptional network that controls the development and function of pDCs from bone marrow to peripheral tissues. The development of pDCs from multiple lineages requires the transcription factors PU.1, CBFβ, IRF8, and TCF3. The primary regulator of pDC development is E2-2, controlled by a network of transcription factors, including BCL11A, ZEB2, and ID2. E2-2 also controls the expression of IRF8 via binding to the *Irf8 + *41kb enhancer region at the CDP stage, possibly through complex formation with other transcription factors such as MTG16. The function of SPIB is to retain immature pDCs in the bone marrow, while RUNX2 expression promotes the egress of pDCs from the bone marrow. Type I IFN production, a significant function of pDCs is mainly controlled by IRF5, IRF7, and IRF8. Other transcription factors, such as E2-2, RUNX2, SPIB, NFATC3, and CXXC5, can directly control IRF7 expression and regulate type 1 IFN production. **(D)** moDCs develop from Ly6C^hi^ monocytes under the control of several transcription factors, including KLF4, MafB, and PU.1, as well as low levels of IRF8. The final differentiation of moDCs also requires the activity of IRF4, ETV6, and ETV3. Arrows indicate positive regulation, while bars indicate negative regulation.

cDC1 differentiation is intimately linked to optimal expression of IRF8 which is tightly regulated by the spatio-temporal coordinated action of key TFs (**Figure 2A**). Indeed, its expression is initiated in early DC progenitors, including Lymphoid Primed Multipotent Progenitors (LMPPs) and is dependent on PU.1-induced chromatin remodelling (61). At the LMPP stage, RUNX and CBFβ induce the activation of the distal +56Kb *Irf8* enhancer that is essential for the initiation of IRF8 expression (62). Further down the path toward DC differentiation the activity of two additional enhancers have been shown to be pivotal in dictating cDC1 vs pDC fate: +41Kb and +32kb *Irf8* enhancers. In progenitors, E protein controls the activation of +41Kb *Irf8* enhancer, which results into the commitment of DC progenitors to the pDC lineage. As alluded to earlier IRF8 expression in progenitors is central for cDC1 differentiation, therefore it has been proposed that the upregulation of ID2 can counteract the action of E protein on the +41Kb *Irf8* enhancer, which results in the activation of the +32Kb *Irf8* enhancer whose accessibility is tightly regulated by BATF3, DC-SCRIPT and IRF8 itself to maintain adequate IRF8 level in pre-cDC1 and cDC1 (46, 63, 64). This key decisional step is also controlled by additional transcription factors, namely ZEB2 (Zinc finger E-box binding homeobox 2) and NFIL3. ZEB2 inhibits ID2 expression of in CDPs thereby promoting pDC differentiation(65, 66). In contrast, NFIL3 acts upstream of ID2 and ZEB2 to control cDC1 differentiation as its binding in CDPs to the -165Kb *Zeb2* enhancer prevents ZEB2 expression in CDPs, promoting the transition from a ZEB2^hi^ID2^lo^ CDPs to ZEB2^lo^ID2^hi^ CDPs (57, 63). This concomitant reduction in ZEB2 expression and increase in ID2 expression drive the differentiation of cDC1s (63). Beyond the important role for IRF8 in controlling DC fate in progenitors, a role for IRF8 in maintaining cDC1 survival has been postulated (67). However, recent studies suggested that rather than being essential for their survival, IRF8 as well as BATF3 control cDC1 identity in fully differentiated cells as their deletion, in both cases, enables the appearance of cDC1-like cells expressing cDC2 features (68, 69).

### 2.2 Key attributes and function of cDC1s

The importance of cDC1s in the immune system has been highlighted by the interrogation of cDC1-deficient mouse models (53, 70). The absence of cDC1s is associated with a reduction in the control of tumor growth (71–76) and impaired control of viral (53) or parasitic (77) infections. The major role of cDC1s in these contexts is inferred from their capacity to activate naïve CD8^+^ T cells. Indeed, cDC1s can confer the 3 signals required for the efficient activation of naïve T cells: 1) the presentation of antigen-derived peptides mainly via cross-presentation, 2) co-stimulatory signals and 3) cytokines.

cDC1 are not only important for the activation of naïve CD8^+^ T cells (78–80) but also for the re-activation of memory CD8^+^ T cells which confer a faster and higher control of secondary infection, as for example in the case of *Listeria monocytogenes* (53). In this setting, cDC1s are the main producer of IL-12 and CXCL9 which facilitate the recruitment and activation of memory CD8^+^ T cells (81). In the tumor context, the production of prostaglandin E2 (PGE2) by tumor cells leads to cDC1 dysfunctionality marked by the downregulation of IRF8, and key effector cytokines such as CXCL9 and IL-12, resulting in poor CD8^+^ T cell tumor infiltration and ultimately in tumor immune evasion (82, 83). Moreover, cDC1s play a major role in licensing CD4^+^ T cells for CD8^+^ T cells activation (84, 85). The cDC1/CD4^+^ T cell interaction through CD40/CD40L signaling increases expression of CD70 and BCL2L11 in the cDC1, allowing an increase in cDC1 survival and the differentiation and expansion of tumor-specific memory CD8^+^ T cells (84, 86, 87).

In addition to their role in the initiation of the CD8^+^ T cell response, cDC1s restrain progenitor of exhausted T (Tpex) cells in the white pulp niche of the spleen in an MHC-I dependent manner. This improves the control of infection by limiting Tpex migration to the red pulp and their differentiation into exhausted T cell (88). How this mechanism can be transposed to the control of tumor growth is still not clear despite evidence of the localization of Tpex in distinct niche in the tumor (89, 90).

## 3 cDC2 development and function

### 3.1 Transcriptional control of cDC2 development

Similar to cDC1s, cDC2s also develop from the CDP, although the transcriptional circuitry controlling cDC2 development is less well understood (**Figure 2B**). As opposed to cDC1s, cDC2s express low amounts of IRF8 and instead highly express IRF4 (Interferon Regulatory Factor 4). Conditional ablation of IRF4 in CD11c^+^ cells has shown impaired, but not the complete loss of cDC2s (91). A potential explanation for the observation that some cDC2s develop in absence of IRF4 could be that the cDC2 population represents a heterogeneous mix of IRF4-dependent and independent subsets. In line with this possibility, a body of work has highlighted a certain degree of diversity within that compartment and the involvement of different TFs (39, 92).

The first report describing cDC2 diversity revealed that the conditional ablation of NOTCH2 (Neurogenic locus notch homolog protein 2) in CD11c-expressing cells resulted in the reduction of ESAM^+^ splenic cDC2s and lamina propria CD103^+^CD11b^+^ DCs (93). Subsequently, the transcription factor KLF4 (Kruppel-like factor 4) was found to be important for the development of ESAM^-^ cDC2s (43, 94). This evidence indicates that NOTCH2 and KLF4 independently control the development of functionally distinct cDC2 subsets (94, 95).

Yet, a study addressing cDC2 heterogeneity at a single cell level has put forward an alternative model to the one proposed here above (39). Brown et al. suggested that cDC2 could be separated instead into T-BET (T-box expressed in T cells) and RORγt (RAR-related orphan receptor gamma) cDC2s, cDC2a and cDC2b respectively. Importantly, in the aforementioned study, neither the expression of *Klf4* or *Irf4* enable the discrimination of cDC2a from cDC2b. Instead, the authors proposed the use of additional cell surface markers, namely CLEC10A and CLEC12A, to separate cDC2a and cDC2b. Interestingly, the interrogation of chromatin accessibility revealed that open chromatin regions in cDC2a showed an enrichment for RBPJ (Recombination signal binding protein for immunoglobulin kappa J region) motifs. As RBPJ is the DNA-binding component of the NOTCH TF complex, this finding is compatible with the earlier reported role for NOTCH2 signaling in controlling cDC2 heterogeneity (39, 93).

In addition to the aforementioned role for ZEB2 in controlling pDC differentiation, a role for ZEB2 in controlling cDC2 development has been shown. However, its function remains controversial as conflicting results have been reported. One study showed that conditional deletion of ZEB2 in *Itgax^cre^Zeb2^fl/fl^* mice led to reduced number of splenic cDC2s (65), but a subsequent study failed to confirm this observation (66). This latest study is somewhat contrasting with the development of a novel mouse model lacking cDC2s and other myeloid lineages (57). In this study, a triple mutation of all three NFIL3-C/EBP sites within the -165Kb enhancer of *Zeb2* ablated its expression exclusively in the myeloid compartment and led to the complete loss of pre-cDC2 specification and mature cDC2 development *in vivo* (57). Whilst the nature of this discrepancy warrants further investigation, these studies also highlighted ZEB2 as a critical regulator of pDC development through its repressive activity on ID2, as well as its important role for monocytes commitment as these 2 populations were strongly affected in this mouse model (57).

### 3.2 Diversity and function of cDC2s in mice and human

Compared to cDC1s, cDC2s appear more efficient in presenting antigens via MHC-II molecules to CD4^+^ T cells (1, 96). However, cDC2s are not equally able to present soluble versus cell associated antigens. CD4^+^ T cell proliferation in response to soluble antigen was unperturbed in mice lacking cDC1s (*Xcr1^DTR^* mice or *Batf3^-/-^* mice), demonstrating that cDC2s compensate for the lack of cDC1s in this setting (53, 97). In contrast, cDC2s are far less efficient than cDC1s in the uptake and processing of cell-associated antigens, and thus display a limited capacity to prime CD8^+^ T cells through this route (98).

As alluded earlier, mice lacking IRF4 were originally used to define the function of cDC2s (91). These studies led to define a key role for cDC2s in the regulation of Th2 and Th17 immune responses aiming to eliminate extracellular pathogens (*Nippostrongylus brasiliensis*) and parasites (*Aspergillus fumigatus*), respectively (91, 99). At that time, it remained unclear how cDC2s could direct such distinctive responses. Some clarification for this division of labor came from studies highlighting the distinct roles for NOTCH2 dependent and KLF4 dependent cDC2s. For example, in the gut NOTCH2-dependent cDC2s were the critical source of IL-23 that were required for clearance of extracellular pathogens such as *Citrobacter Rodentium* though the induction of a Th17 biased immune response (100, 101). In addition, NOTCH2-dependent splenic cDC2s were required to promote T follicular helper (T_FH_) cell and germinal center (GC) B cell formation in response to *Listeria monocytogenes* (102, 103). In contrast, it was found that conditional deletion of *Klf4* in DCs was detrimental for Th2, but not Th17, immune responses in mice (94). In line with the above, a STAT6/KLF4 dependent CD11b^low^ cDC2 population localized in the skin has been shown to mediate Th2 immune responses (43).

cDC2s are also important for the T cell response to viral infection. Following PV (single-stranded RNA pneumonia virus) infection, cDC2s can acquire a hybrid phenotype characterized by increased IRF8 expression and the capacity to prime both CD4^+^ and CD8^+^ T cells. The acquisition of these cDC1-like properties by cDC2s was dependent on the signaling via Toll-like receptors and the type 1 interferon receptor (104). Additionally, the induction of T_FH_ cell differentiation was dependent on the presentation of viral antigens at the T/B border by migratory cDC2s (102). Furthermore, LN resident cDC2s are strategically positioned to capture the influenza A virus (105) and other blood born antigens (106) resulting in the rapid initiation of T cell responses, independent of migratory DCs influx. While moDCs were also reported to activate T cells under similar conditions (107, 108), some studies have suggested that inflammatory cDC2s can acquire moDC like features, such as the expression of MAR-1 and CD64, and the moDCs will express cDC2 signature genes including CD11b and CD172a, suggesting that the antigen presentation capacity of moDCs may actually be due to contamination by inflammatory cDC2s (104, 105). In agreement with this conclusion, the use of CD26 as an additional marker to differentiate inflammatory cDC2 from moDCs, highlighted the limited antigen presentation capacity of CD26^-^ moDCs (104).

Collectively, these studies highlight the functional specificities of the various cDC2 subtypes within different organs. Deciphering the molecular mechanisms underpinning this diversity is a prerequisite to define the role of these different subsets of cDC2s in initiating adaptive immune responses in the context of pathogens, virus infection and tumor clearance, as this knowledge will provide a rational framework for their use in clinical settings.

## 4 DC3: a unique DC subtype or the DCs with different cells state?

The application of single-cell RNAseq technology to DCs has led to many reports of novel DC subtypes (38, 40, 92, 109). The use of different annotation strategies to define populations with otherwise very similar transcriptomic features has created a good deal of confusion in the field (110). The status of the DC3 population represents an example of this issue.

DC3s were initially identified in the blood of humans through single-cell RNA sequencing (38). The subsequent studies phenotypically characterized the DC3 population as CD163^+^CD14^+^ DCs that accumulate in the blood of patients with systemic lupus erythematosus (SLE) (40). DC3s display an intermediate phenotype and function between cDC2s and monocytes and are characterized by low expression of IRF8 (111). Unlike cDC1s and cDC2s, the development of DC3s relies on GM-CSF, but not FLT3L, and it is developmentally independent of the CDP (92). Functionally, these cells have been proposed to promote the differentiation of naïve CD8^+^ T cells into tissue-homing CD103^+^ T cells (92).

The AXL^+^ DC subpopulation was also reported in the blood of humans, alongside the DC3 population, displaying an intermediate phenotype between cDC2s and pDCs (38). This population was characterized by the expression of Siglec6 and AXL. Similarly, in mice, transitional DCs (tDCs), also referred to as “pDC-like” cells, with characteristics spanning between cDC2s and pDCs, were observed during steady-state and influenza infection, and appear to be the equivalent to the AXL^+^ DCs in humans (109). It has been recently proposed that these “pDC-like” cells are pre-cDC2s and require KLF4 for both their development and function (112).

Other similar single-cell transcriptomic studies have identified another DC population that exhibits an “activated” DC phenotype and is referred to as “DC3” in both mouse and human (113). This DC population lacks the canonical cDC1s and cDC2s gene signature but expresses the matured cDC1 and cDC2 signatures (113). Similar population have also been described as CCR7^+^LAMP3^+^ DCs, Mreg DCs or ISG^+^ DCs within tumors (114–116). It is important to note that these “activated” DC populations represent developmental states of both cDC1s and cDC2s and therebefore they are not to be confounded with CD163^+^CD14^+^ DCs (DC3s) reported by Dutertre, Cytlak, Bourdely and Villani et al. Currently, it is recommended to designate this “activated” DC population as “CCR7^+^ DCs” due to the consistent detection of CCR7, a common marker for DC activation and maturation, in various contexts except ISG^+^ DCs (110, 116).

Sorting out the cellular relationships between the cDC1, cDC2, DC3 and CCR7^+^DCs populations is one of the key goals for the DC field moving forward. Regardless of their development origins, identifying the environmental cues and the molecular mechanisms driving DC3 and CCR7^+^ DC phenotype and functional attributes also warrants further investigation.

## 5 pDC development and function

### 5.1 pDC ontogeny

pDCs are a distinct cell type first identified through their capacity to rapidly produce large amounts of type I interferons (IFNα/β) (117–120). Whether pDCs developed from lymphoid or myeloid progenitors has remained a controversial question for more than two decades (34, 121). Similar to the development of cDCs, Flt3 signaling is required for optimal pDC development (122). Yet as opposed to cDCs, that can only originate from the myeloid progenitors, Flt3^+^ CMPs, CDPs and CLPs have all been shown to retain pDC potential both *in vitro* and *in vivo* following adoptive transfer (44, 45, 48, 49, 123–125). These findings led to the concept that pDC have a dual origin: myeloid and lymphoid (**Figure 2C**). However, the myeloid origin of the pDCs is being disputed by different groups (35, 36, 125, 126). This issue has been revisited with IL-7R^+^ lymphoid progenitors being proposed to be the main source for pDCs *in vivo* (126*)*. A predominantly lymphoid origin for the pDCs is also supported by their expression history of the recombination activating gene 1 (*Rag1*) and the rearrangement of the D-J regions of the *Igh* locus (125, 127). In an effort to distinguish the properties of myeloid- vs lymphoid-derived pDCs, it was found that the myeloid-derived Zbtb46^+^ pDCs have a distinct transcriptome that resulted in them being more efficient than lymphoid-derived pDCs in their capability to present antigens to T cells (125). While this study is accordance with earlier reports pointing to the dual origin of pDC (127), these findings were subsequently challenged by a study that proposed that a CD115^-^ Ly6D^+^ lymphoid progenitors are the sole source of pDCs *in vivo* (126). Crucially, the definition of a lymphoid or myeloid origin of pDCs largely depends on the markers used to track the development history of pDCs. For example, Dress et al. used CD2 as a lymphoid lineage marker to trace the development history of pDCs, and conclude that the pDCs are of lymphoid origin (41, 126). However, CD2 expression is not restricted to the lymphoid lineage as 20% of the cDC are fate mapped in the hCD2-iCre^+/–^R26-stop-EYFP^+^ mouse model (128), thus this model cannot completely rule out the participation of myeloid biased progenitor to the pDC pool. Adding to that, clonal tracing of HSC and CX3CR1^+^ progenitors using *FlipJump* system and single-cell transcriptome and phenotype analysis (CITE-seq) suggested that cDCs and pDCs share a common progenitor (129). Further characterization of the pDCs specific transcriptional program will be helpful to improve our understanding of pDC ontogeny and the heterogeneity of this population.

### 5.2 Transcriptional control of pDCs development

The development of pDCs requires the TF E2-2 (E protein encoded by *Tcf4*) (**Figure 2C**). E2-2 deficient mice die *in utero*, but transfer of *Tcf4*^-/-^ fetal liver cells into irradiated WT recipients results in the complete loss of pDCs from the BM and all peripheral lymphoid organs, but has no impact on the development of other myeloid or lymphoid cell types (33). E2-2 is a member of the basic helix-loop-helix superfamily of TFs that has long (E2-2_L_) and short (E2-2_S_) isoforms (130). E2-2_S_ is expressed in all hematopoietic progenitors and different types of mature immune cells, but E2-2_L_ is preferentially expressed in pDCs and binds to the pDC specific 3’ enhancer of *Tcf4* to maintain E2-2s expression via a positive feedback loop (130). E2-2s expression initiates in HSCs and is further upregulated during pDC development. E2-2s forms a complex with Mtg16 (myeloid translocation gene on chromosome 16) to directly control the expression of key genes involved in pDC development and function, including CCR9, TLR9, Bst2 and B220 (131). In DC progenitors, ID2 as an E protein inhibitor binds E2-2s preventing its binding to DNA, and thereby inhibits their pDC potential (63). In contrast, ZEB2 expression in progenitors prevents ID2 expression, enabling E2-2s to promote pDC development. In line with the above, constitutive deletion of -165kb *Zeb2* enhancer featuring a cluster of E box motifs, results in lack of ZEB2 expression, increased ID2 expression that prevents pDC differentiation (132). Thus, the coordinate action of E2-2_L_, E2-2s, ID2 and ZEB2 dictates pDCs development at steady state.

Other TFs have been implicated in the cellular fate of BM progenitors. PU.1 is highly expressed in myeloid and lymphoid BM progenitors, but its expression level is substantially reduced following the commitment of progenitors to the pDC lineage (55, 122, 133, 134). High expression of PU.1 in cDC was shown to be essential to maintain their identity as PU.1 deficient cDCs gained pDC like features (55). Thus, it is conceivable that downmodulation of PU.1 in progenitors constitutes a key instrumental step in allowing pDC differentiation (135). In line with this, the expression of PU.1 is negatively regulated by BCL11A (B-cell chronic lymphocytic leukaemia/lymphoma 11A), a critical regulator of pDC development (136). Adding to that, loss of PU.1 in CD11c^+^ cells resulted in an increased differentiation of progenitor toward the pDC lineage, although PU.1 deficient pDCs were dysfunctional, as IFNα production was reduced in PU.1 deficient pDCs (55). In contrast to the down-modulation of PU.1 following pDCs commitment, IRF8 expression is increased markedly during pDC development (67). Thus, it is somewhat surprising, that IRF8 deficiency in CD11c^+^ cells has no impact on the development of pDCs. This is in fact due to a compensatory mechanism provided by IRF4 as double knockout mice lack pDCs (67). Although IRF8 is dispensable for pDC differentiation, it is essential for their IFNα production, thus indicating a nonredundant role for IRF8 in controlling pDC function.

Spi-B is another ETS family TF that is highly expressed in pDCs (137). In contrast to the decreased PU.1 expression following pDCs development, Spi-B expression is substantially increased from progenitors to mature pDCs. Germline deletion of *SpiB* results in decreased pDC numbers in the BM but their numbers are increased in peripheral organs (138). These data suggests that Spi-B is dispensable for pDC differentiation but a critical regulator of pDC homeostasis. Having said that, its role and its mode of action in pDCs remains under investigated. In contrast to BM, the TF RUNX2 (RUNX family transcription factor 2) promotes pDC their egress, as germline ablation or tamoxifen induced deletion of RUNX2 result in reduced number of peripheral pDCs, whilst RUNX2 is dispensable for their differentiation in the BM (139, 140). Two mechanisms were proposed. Sawai et al. showed that RUNX2 was required for the expression of chemokine receptors on the cell surface of pDCs including CCR2 and CCR5 that were required for the migration of pDCs from BM into the periphery in response to their ligands (139). In contrast, Chopin et al. demonstrated that RUNX2 deficiency resulted in increased expression of CXCR4, a key chemokine receptor associated with BM tropism (140). Spi-B and RUNX2 are not only critical regulators of pDC homeostatic in the periphery but also have been both shown to be critical for IFNα production by pDC, though the regulation of *Irf7* (138, 140).

BCL11A is a zinc-finger TF and is known to regulate lymphoid development (141). Both BCL11A and PU.1 control *Flt3* expression in early hematopoietic progenitors (142), which is required for pDC development and their homeostasis. ChIP-seq data showed that BCL11A bound to the *Tcf4* proximal promoter and knockdown of BCL11A strongly reduced E2-2 expression (136). Interestingly, downregulation of *Bcl11a* occurred after *Tcf4* deletion in BM derived pDCs (143), indicating a positive feedforward loop between BCL11A and E2-2 in controlling pDC development.

IKAROS (encoded by *IKAROS Family Zinc Finger 1* (*Ikzf1*)) is a zinc-finger DNA-binding protein that homo- or hetero-dimerizes with other IKAROS family members to suppress the gene expression. IKAROS prevents premature cDC gene expression in CDPs and promotes pDC development (144, 145). The relationship between IKAROS with other TFs that control the development and function of pDCs has not been studied.

Collectively, these studies have revealed a dynamic TF network that regulates the development of pDCs within the hematopoietic system. These studies also highlight a critical point in the current debate about whether pDCs and cDCs share a common ancestor. These findings suggest that the lineage trajectories of DCs are dictated by mutual antagonism between transcription factors (E2.2/ZEB2 vs ID2/NFIL3 or PU.1 vs BCL11A), thus inferring a close relationship between pDCs and cDCs.

### 5.3 The function of pDCs in mouse and human

Unlike cDCs, pDCs have limited capacity to present antigens. Instead, their key feature is the rapid production of type I IFNs (IFNα/β) after exposure to the ligands for TLR7 (recognize ssRNA) and TLR9 (recognize CpG), especially after the viral infection (33, 146, 147). The early production of type I IFNs by pDCs initiates the anti-viral gene expression program in many cell types and promotes the expansion of NK cells and virus specific CTLs for viral clearance (146, 147). This type I IFN production results in the apoptosis of activated pDCs, potentially limiting the scale of inflammatory response and preventing pathology associated with an overly active anti-viral immune response (148). This control appears important as aberrant type I IFN production by pDCs is strongly linked to the development of autoimmune diseases like SLE and systemic sclerosis in both mouse models and human (149, 150).

## 6 moDCs development and function

### 6.1 Transcriptional control of moDCs development

The ambiguous nature of moDCs has hampered our capacity to define some of the key TFs associated with their differentiation. Lineage tracing experiments have demonstrated that moDCs derive from a separate myelopoiesis branch distinct from the one producing cDCs and pDCs (151). In contrast to the requirement of high dose IRF8 for cDC1 development, moDCs develop in a relatively low concentration of IRF8. This expression of IRF8 is driven by *Irf8 + *56kb enhancer whose activation is controlled by RUNX-CBFβ (62). The differentiation of Ly6C^+^ monocytes into moDCs or macrophages is controlled by the TFs IRF4 and MafB (MAF BZIP Transcription Factor B), and PU.1 (**Figure 2D**) (152–154). The differentiation of mouse monocyte into moDCs in presence of GM-CSF and IL-4 requires IRF4. In its absence, the cells differentiate into macrophages (155). It also had been reported that MafB expression will push the human monocytes into the macrophage pathway, while high concentration of PU.1 will suppress MafB and thus promote differentiation into moDCs (152, 156). Apart from PU.1, a most recent study found that ETV3 and ETV6 are able to repress macrophages development potential in monocytes by suppressing MafB expression in both mouse and human (154). Thus, moDCs use a distinct repertoire of TFs compared to those that promote cDC development.

### 6.2 The function of moDCs

Monocytes represent a major cell population in the circulation, from which they are recruited into the tissues by inflammatory cues and give rise to both macrophage and moDCs. Normally, monocytes express Ly6C and macrophage colony stimulating factor receptor (M-CSFR/CD115) and respond to GM-CSF (157). The moDCs can be easily confounded for cDCs in tissues as they share a variety of cell surface markers including the “canonical DC markers” MHC-II and CD11c, as well as the cDC1 marker CD24 and the cDC2 marker CD172a (158). In addition to sharing cDC phenotypic features, moDCs can present antigen to both CD4^+^ and CD8^+^ T cells. Notably, moDCs can cross-present antigen released from certain microorganisms to CD8^+^ T cells under acute inflammation condition and might replace some (41), but not all anti-infection functions of cDCs (77). As per their cDCs counterpart, moDCs express costimulatory molecules that support the differentiation of CTLs (159) and present antigen directly to CD4^+^ T cells promoting their differentiation into Th17 cells (160). Furthermore, moDCs are strong producers of proinflammatory cytokines including IL-1β, TNFα, IL-23 (161), and IL-12 in cancer (162). Collectively, although moDCs arise from a distinct myeloid branch compared to cDCs, both subsets share a substantial number of overlapping phenotypic and functional characteristics after activation.

## 7 Concluding remarks

Recent advances in the field of DC research have provided new insights into the heterogeneity and functional diversity of DC subsets. Studies on the transcriptional regulation of DC development and function have led to the identification of key TFs and their targets that shape the transcriptome and function of DCs. In-depth phenotyping of DCs has also identified novel DC subtypes, such as DC3, highlighting the need for continued investigation into the ontogeny of DCs. While much progress has been made, much is still to be learned about the intricate connections between different TFs and their doses regulating the differentiation and activation of DCs.

Whilst we try to build a comprehensive map of the transcriptional network governing DC heterogeneity, which will be essential for their clinical application, there is an urgent need to understand how DC functionalities, independently of their origin, are shaped by environmental signals. To fulfill the long-recognized potential of DC based therapy to treat malignancies, we believe that an in-depth characterization of the signals that drive their diversity and a better under understanding of the environmental cues that shape their functional attributes is urgently required.

## Author contributions

SZ, CA and MC contributed to original draft preparation. SZ contributed to the figures. SN, MC, CA and SZ contributed to review and editing. All authors contributed to the article and approved the submitted version.
